# American Medical Association—Means for Its Improvement

**Published:** 1872-12-15

**Authors:** 


MEDICAL EXAMINER.

*A Semi-Monthly Journal of Medical Sciences.*

EDITED BY

N. S. DAVIS, M. D., AND F. H. DAVIS, M. D.

Chicago, December 15th, 1872.

### EDITORIAL.

### AMERICAN MEDICAL ASSOCIATION. —MEANS FOR ITS IMPROVEMENT.

Having in two or three preceding numbers of THE EXAMINER, noticed the principal objections to the present organization of the National Association and the suggestions made for its improvement, we feel under obligations to give the results of our own observation and reflection upon the subject. We by no means agree with those who represent the Association as a failure under its present organization. On the contrary, it would not be difficult to show that its influence has been, and still is, very great in all the directions in which it was originally designed to act. That its influence has greatly increased the number and efficiency of the State and local medical societies — that its annual meetings have greatly advanced both the social and scientific intercourse of members of the profession throughout the whole country — that this intercourse has greatly diminished sectional prejudices, and substituted in their place personal friendships — and finally, that it has been largely instrumental in causing the extension of the lecture terms, and the curriculums of study in nearly all the medical schools, and the actual introduction into some of them of a graded or systematic and more complete system of medical teaching, no one who takes the trouble to examine the historical facts, can reasonably entertain a doubt. That there has been no falling off in the number of those who seek its membership and participate in its proceedings, or strive for its honors, the last annual meeting will abundantly show. And if its present organization and by-laws, or rules of action, were fully understood, and the several sections induced to carry out more fully the objects for which they were created, it would result in practically obviating nearly all the important objections that have been urged against the Association. Yet it must be acknowledged that in so large a body, gathering its members from sections so wide apart, and holding its sessions so brief a period, it is almost impossible to have the majority of its members present at any annual meeting, sufficiently familiar with the practical working of its several parts, to make the general result harmonious and satisfactory. The annual meetings of the Association, as at present organized, are too large and too deficient in true representative character, to be reliable and efficient for deliberate legislation on matters of medical polity, or for the direct promotion of medical science. They are well adapted, only, for listening for two or three hours of the morning of each day to well prepared addresses or reports on the progress made in the different departments of medical science and art, and then devoting the remainder of the day to sight-seeing and social intercourse. If such changes in the present organization could be made, as would secure the three following objects, it would undoubtedly constitute a very great improvement.

First, the adoption and enforcement of a fair and uniform standard of medical attainments as a condition of membership in the Association.

Second, the retention of, at least, biennial mass meetings of the membership, which shall be devoted exclusively to the hearing and discussion of addresses on the progress of the different departments of medicine, and to free social intercourse.

Third, the organization of a general council composed of not more than two members from each State, elected in such a manner as to make them strictly representative in character, and holding their office two years. Such council should meet annually, and to it should be entrusted all the strictly business matters of the Association. Such a council would constitute a compact, intelligent representative body, capable of being so organized into sections and committees as to secure full and deliberate attention to all the important interests of the profession. At the same time, the biennial mass meetings of the membership in the several cities in different parts of the Union, for the purposes stated in a preceding paragraph, would not only retain all the benefits of the present annual meetings, but allow of a great improvement in the social advantages. The first object, namely, the adoption and enforcement of a uniform standard of qualifications for membership in the Association, instead of having members elected annually by all sorts of medical societies, colleges, hospitals, dispensaries, infirmaries, etc., would require the establishment of one or more boards of censors in each State, with strict rules for their guidance, by whom all applicants for admission to membershlp in the Association should be examined; and those found qualified should receive a certificate or diploma of membership in both the National Association, and State Society of the State in which they live.

And after such boards of censors have been organized and ready for action, there should be no other mode of gaining admission for new members, except through actual examination of such boards. This plan would cut off the whole of those really self-appointed delegates, a large part of whom go to the annual meetings with some private axe to grind, or some specialty to advertise; and what is of far more importance, the standard of qualifications adopted by these boards of censors and the certificate or diploma given by them, would soon come to be recognized as the only legitimate mode of entering fully the ranks of the profession.

Whether the details of a plan for making the changes suggested, could be so arranged as to work well, practically, and secure general approval, remains to be seen. To preserve and foster the connection between the National Association and the several State Medical societies, the members of the General Council of the National Association, consisting of two from each State, should be elected by the State societies; while, to secure as much uniformity of action as possible by the examining boards, the nomination of these, together with the rules governing them, should be made by the General Council. An ample basis of membership could be secured at once, by issuing certificates or diplomas of membership to all who should be members in good standing at the time of adopting the new arrangement, and subsequently admitting no new members, except through the examination and approval of the proper Boards of Censors. This would effectually stop all questions as to the right of this or that institution, college, or society, to representation in the association, and place college faculties, hospital staffs, specialists with infirmaries, etc., on the same footing with all other members of the profession. There would be left but one door of entrance to membership in the national organization for all classes; and it is very plain that the standard of education required to pass that door, would soon become *the only* recognized standard for the whole profession.

---

Pustulation with Tartar-Emetic as a Substitute for Vaccination.—Dr. B. S. Woodworth, of Fort Wayne, one of the oldest and most eminent physicians in Indiana (*Am. Practitioner*, Sept., 1872), writes that his theory—which he has held for thirty-four years—is, that pustulation by tartar-emetic is as good a preventive of small-pox as perfect vaccination. He was led to this theory from a series of papers, in 1838–9, published in the Boston *Medical and Surgical Journal*, in which the almost exact resemblance of tartar-emetic pustules and vaccinia was mentioned. This fact, he adds, is familiar to every physician. Not only is the similarity of the two perfect at their full development, but the successive stages are also alike. He asks: Why may not this artificial pustulation be just as good a preventive as vaccination, since the same process has been gone through with, and the same molecular changes? And certainly there can be nothing more mysterious in the one case than in the other. Some five or six years after he had first entertained this view, an article in the London *Lancet*, by a German author, advanced the same theory, and, he believes, verified by cases. Dr. Woodworth suggests that experiments be made in hospitals, to prove his theory; and closes his communication with these words: "I almost regret that I was ever vaccinated, for I would be willing to run the risk in proving that which I believe true."—*Med. Record.*

Contagiousness of Purulent Secretions.—Dr. Hiller, says the London *Lancet*, in an inaugural dissertation on this subject, maintains the truth of the old view that all purulent secretions are contagious when applied to healthy mucous membrane. He states that pus taken from the vagina of a bitch in which inflammation had been excited by the injection of ammonia produced pyorrhœa in the urethra of a dog into which it was injected, though not before the latter had been mechanically irritated. If no mechanical irritation were employed, no catarrh occurred. The pyorrhœa, both in the male and female animal, appeared to be closely analogous in its nature to gonorrhœa, since it spread in the former to the posterior part of the urethra, and in the latter to the mucous membrane of the uterus. M. Hiller holds that the same obtains in the case of the eye, and that all purulent discharges applied to the conjunctiva are capable of exciting inflammation in it. It is certain, however, that the matter from an inflamed lachrymal sac, or from an abscess of the lid, may freely enter the eye without producing a purulent discharge.—*Lancet for August* 31, 1872. *Mich. University Med. Journal.*

Treatment of Uterine Hæmorrhage by Sulphate of Quinine.—MM. Gueneau de Mursy and Bartheg, have resorted, for several years past, to the use of this remedy in the treatment of metrorrhagia, with marked success. It is principally in hæmorrhages which occur several days after delivery and are accompanied by fever, with daily exacerbations, that they have found its use to be of most value; and in several cases it has given relief when used as a *dernier resort*, after ergot and other hæmostatics failed.

In order to obtain the desired results, it is necessary to give the remedy in sufficiently large doses. The gentlemen above mentioned have used it at the rate of eight grains every two hours. Sometimes it is not necessary to continue the remedy beyond a single day, but it is generally prudent to continue it for several days, even though the hæmorrhage should have ceased.

Mental Disease Prize.—M. Tarlet has made a bequest of 10,000 francs to the Paris Academy of Medicine, for the purpose of founding a prize on mental and nervous diseases.

